# Elevated leptin levels induce inflammation through IL-6 in skeletal muscle of aged female rats

**DOI:** 10.1186/s12891-019-2581-5

**Published:** 2019-05-10

**Authors:** Ryo Tazawa, Kentaro Uchida, Hisako Fujimaki, Masayuki Miyagi, Gen Inoue, Hiroyuki Sekiguchi, Kosuke Murata, Ken Takata, Ayumu Kawakubo, Masashi Takaso

**Affiliations:** 1 Department of Orthopedic Surgery, Kitasato University School of Medicine, 1-15-1 Minami-ku, Kitasato, Sagamihara City, Kanagawa 252-0374 Japan; 2 Shonan University of Medical Sciences Research Institute, Nishikubo 500, Chigasaki City, Kanagawa 253-0083 Japan

**Keywords:** Leptin, Interleukin-6, Intermuscular adipose tissue, Inflammation

## Abstract

**Background:**

Chronic inflammation with aging contributes to sarcopenia. Previous studies have suggested that the accumulation of adipose tissue in skeletal muscle, referred to as intermuscular adipose tissue (IMAT), increases with age and is associated with inflammation. However, the mechanism governing ectopic inflammation in skeletal muscle due to aging is not fully understood. Leptin, an adipocytokine derived from adipose tissue, is an important mediator of inflammatory processes. We examined changes in leptin levels with age and whether leptin contributes to ectopic inflammation.

**Methods:**

To evaluate ectopic inflammation in skeletal muscle, we measured alterations to the expression of inflammatory cytokine genes (*Il1b, Il6*, and *Tnfa*) and muscle break down-related gene (*MuRF1* and *Atrogin1*) in the quadricep muscles of young (10 weeks) and aged (48 weeks) female rats using quantitative reverse-transcription polymerase chain reaction (Q-RT-PCR). Histological examination was performed to identify the extent of IMAT. *Leptin* mRNA and leptin protein expression were examined using Q-RT-PCR and enzyme-linked immunosorbent assay, respectively. The effect of leptin on the mRNA expression of *Il1b*, *Il6*, and *Tnfa* in quadricep muscle-derived cells was also examined by stimulating the cells with 0 (control), 1, or 10 μg/mL rat recombinant leptin using Q-RT-PCR.

**Results:**

Aged rats had significantly higher *Il6, MuRF1, and Atrogin1* but not *Il1b* and *Tnfa,* expression and greater levels of IMAT in their quadricep muscles than young rats. Aged rats also had significantly higher *leptin* expression and leptin protein concentration in their quadricep muscles than young rats. The addition of exogenous leptin to quadricep muscle-derived cells significantly increased the gene expression of *Il1b* and *Il6* but not *Tnfa*.

**Conclusions:**

Our results suggest that elevated leptin levels due to aging cause ectopic inflammation through IL-6 in the skeletal muscle of aged rats.

**Electronic supplementary material:**

The online version of this article (10.1186/s12891-019-2581-5) contains supplementary material, which is available to authorized users.

## Background

Sarcopenia is the reduction in skeletal muscle mass and function with age, and is a major public health concern. The sarcopenia-related morbidity rate is 5–13% in 60- to 70-year-old individuals, and 11–50% in those above 80 years old [1]. Sarcopenia can lead to a rise in the incidence of falls and the risk of fractures in the elderly, and is therefore linked to physical disability, and increased mortality, morbidity, and health care costs [2, 3]*.* Although a number of factors are implicated in the pathophysiology of sarcopenia, its pathophysiology remains elusive.

Proinflammatory cytokines associated with skeletal muscle metabolism contribute to sarcopenia [4–9]*.* A previous study reported that older individuals with sarcopenia had higher levels of serum interleukin (IL)-6 than those without sarcopenia [7]. IL-1β concentrations increase in inflammatory conditions, and elevated levels of IL-1β inhibit myoblast differentiation [8, 9]*.* However, the mechanisms governing the abnormal levels of IL-1β and IL-6 expression in skeletal muscle due to aging is not fully understood.

The accumulation of adipose tissue in skeletal muscle, referred to as intermuscular adipose tissue (IMAT), increases with age. Adipose tissue produces several adipocytokines and regulates inflammatory conditions [10]. Leptin, one such adipocytokine derived from adipose tissue, is an important mediator of inflammatory processes [11]. Previous studies have shown that leptin has pro-inflammatory properties and upregulates *Il-6* expression in human synovial fibroblasts [12] and rat microglia [13]. Leptin also stimulates *Il1b* expression in human chondrocytes [14] and rat microglia [15]. We hypothesized that IMAT-derived leptin may be differentially expressed in the muscle with age and may regulate the expression of inflammatory cytokines.

Here, we investigated the expression of leptin and inflammatory cytokines with age and the relationship between leptin and inflammatory cytokines in rat muscle.

## Methods

### Animals

This study used female Sprague-Dawley (SD) rats obtained from Charles River Laboratories Japan, Inc. (Yokohama, Japan). Rats were fed a commercial pelleted diet (CRF-1, Oriental Yeast Industry, Tokyo). All experimental protocols were in accordance with the guidelines of the Animal Ethics Committee of Kitasato University (Permission number: 2018–085).

### Quantitative reverse-transcription polymerase chain reaction (Q-RT-PCR) analysis

Preliminary experiments using rats aged 10, 24, 48, and 96 weeks indicated that leptin mRNA expression in 48- and 96-week-old rats was significantly higher than that in 10-week-old rats (Additional file 1: Figure S1). However, a previous study reported that SD rats developed a tumor early or late in life, over the age range of 494 to 798 days (approximately 71 to 114 weeks) at the time of first tumor observation [16]. Therefore, to evaluate changes in cytokine expression due to aging, SD rats were divided into two age groups: the young group (10 weeks) and aged group (48 weeks) (*n* = 10 each). Animals were anaesthetized first with isoflurane following by an intramuscular injection of a mixture comprising medetomidine, midazolam, and butorphanol tartrate into the upper limb. Blood was removed to prevent contamination with blood components before sacrificing the rats by cervical dislocation. Bilateral quadricep muscles were removed without the fascia and washed with phosphate buffered saline solution (PBS). TRIzol (Invitrogen, Carlsbad, CA, USA) was used to extract total RNA from the quadricep muscles, based on the manufacturer’s protocol. The RNA formed the template for first-strand cDNA synthesis, which was performed with SuperScript III RT (Invitrogen) in a reaction (final volume, 25 μL) comprising 2 μL cDNA, a specific primer set (0.2 μM final concentration), and 12.5 μL SYBR Premix Ex Taq (Takara, Shiga, Japan). Primers for *Il1b, Il6, Tnfa, MuRF1, Atrogin1,* and *leptin* were designed using Primer Blast and made by Hokkaido System Science Co., Ltd. (Sapporo, Japan). The primer sequences are listed in Table 1. The primer-amplified products were confirmed for specificity using melt curve analysis. Q-RT-PCR was conducted on a CFX-96 Real-Time PCR Detection System (Bio-Rad, Hercules, CA, USA). The Q-RT-PCR protocol was as follows: initial denaturation at 95 °C for 1 min, and 40 cycles of 95 °C for 5 s and 60 °C for 30 s. mRNA expression levels of inflammatory cytokine (*Il1b, Il6, Tnfa)*, muscle breakdown-related marker (*MuRF1*, *Atrogin1*) and *leptin* in the quadricep muscles were determined by normalizing to that of glyceraldehyde-3-phosphate dehydrogenase (*Gapdh*) using the delta-delta Ct method. Relative expression was calculated using the mean of all control samples (samples from quadriceps muscles from the young group or vehicle-treated quadriceps muscle-derived cells in vitro).

**Table 1:** Sequences of the primers used in this study

Gene	Direction	Primer Sequence (5′–3′)	Product Size (bp)
*Il6*	F	CCAGTTGCCTTCTTGGGACT	224
	R	TCTGACAGTGCATCATCGCT	
*Il1b*	F	CCTCGTCCTAAGTCACTCGC	156
	R	GCAGAGTCTTTTTGACCCTCCT	
*Tnfa*	F	CTCTTCTCATTCCCGCTCGT	104
	R	GGGAGCCCATTTGGGAACTT	
*MuRF1*	F	TGCAAGGAACACGAAGACGA	170
	R	ACAAGGAGCAAGTAGGCACC	
*Atrogin1*	F	GGTCTCACGATCACCGACCT	136
	R	TCCACAGTAGCCGGTCTTCA	
*Gapdh*	F	TGC CAC TCA GAA GAC TGT GG	129
	R	TTCAGCTCTGGGATGACCTT	

### Histological evaluation

To investigate the accumulation of adipose tissue in skeletal muscle due to aging, SD rats were again separated into two age groups: the young group (10 weeks) and aged group (48 weeks). After anesthetization with isoflurane following by an intramuscular injection of a mixture comprising medetomidine, midazolam, and butorphanol tartrate, rats were sacrificed by cervical dislocation. Quadricep muscles were removed without the fascia and fixed in paraformaldehyde before embedding in paraffin. The tissue was cut into 3-μm-thick sections and stained with hematoxylin-eosin (HE).

### Enzyme-linked immunosorbent assay (ELISA) for leptin

To investigate changes in leptin protein expression due to aging, ELISA was performed on tissue obtained from rats separated into the same two age groups as the above experiments: the young group (10 weeks) and aged group (48 weeks) (*n* = 10 each).

Quadricep muscles harvested from rats as described above were homogenized in radioimmune precipitation (RIPA) buffer (Wako Pure Chemical Co., Inc., Osaka, Japan) containing an added protease inhibitor cocktail (Roche, Madison WI, USA). Total protein concentration in the solution was ascertained by the bicinchoninic acid (BCA) assay (Thermo Fisher Scientific, Rockford IL, USA) and leptin protein concentration by a rat leptin ELISA kit (R&D Systems, Inc., Minneapolis MN, USA).

### Effect of leptin on *Il1b, Il6* and *Tnfa* expression in quadriceps muscle-derived cells

Our preliminary experiments showed that there was no difference between young and aged rats in response to leptin. Dose of leptin was determined based on previous studies [13, 17]. Ten-week-old SD rats were used for this experiment. Quadricep muscles were removed bilaterally as described above and digested with type I collagenase overnight at 37 °C to extract muscle cells. The cells were cultured in α-MEM supplemented with 10% fetal bovine serum in six-well plates for 1 week at 37 °C in a 5% CO_2_ incubator. After 1 week of incubation, cells were confluent on the wells. Subsequently, recombinant rat leptin (0, 1 and 10 μg/mL) (Biolegend, San Diego, CA, USA) was added, and the cells were stimulated for 24 h. Total RNA was extracted from treated (1 and 10 μg/mL leptin) and control (0 μg/mL leptin) cells, and *Il1b*, *Il6 and Tnfa* expression was ascertained using Q-RT-PCR.

### Statistical analysis

Differences between the two age groups were compared using the Mann-Whitney U test. Differences among treated and control muscle-derived cells were compared using the one-way ANOVA and Tukey multiple comparison’s test. SPSS was used as the statistical software (Version 19.0; SPSS, Inc., Chicago, IL, USA), and *p* < 0.05 was used to indicate statistical significance.

## Results

### Expression of inflammatory cytokines and muscle breakdown-related gene expression in quadriceps muscles

**Fig. 1:**
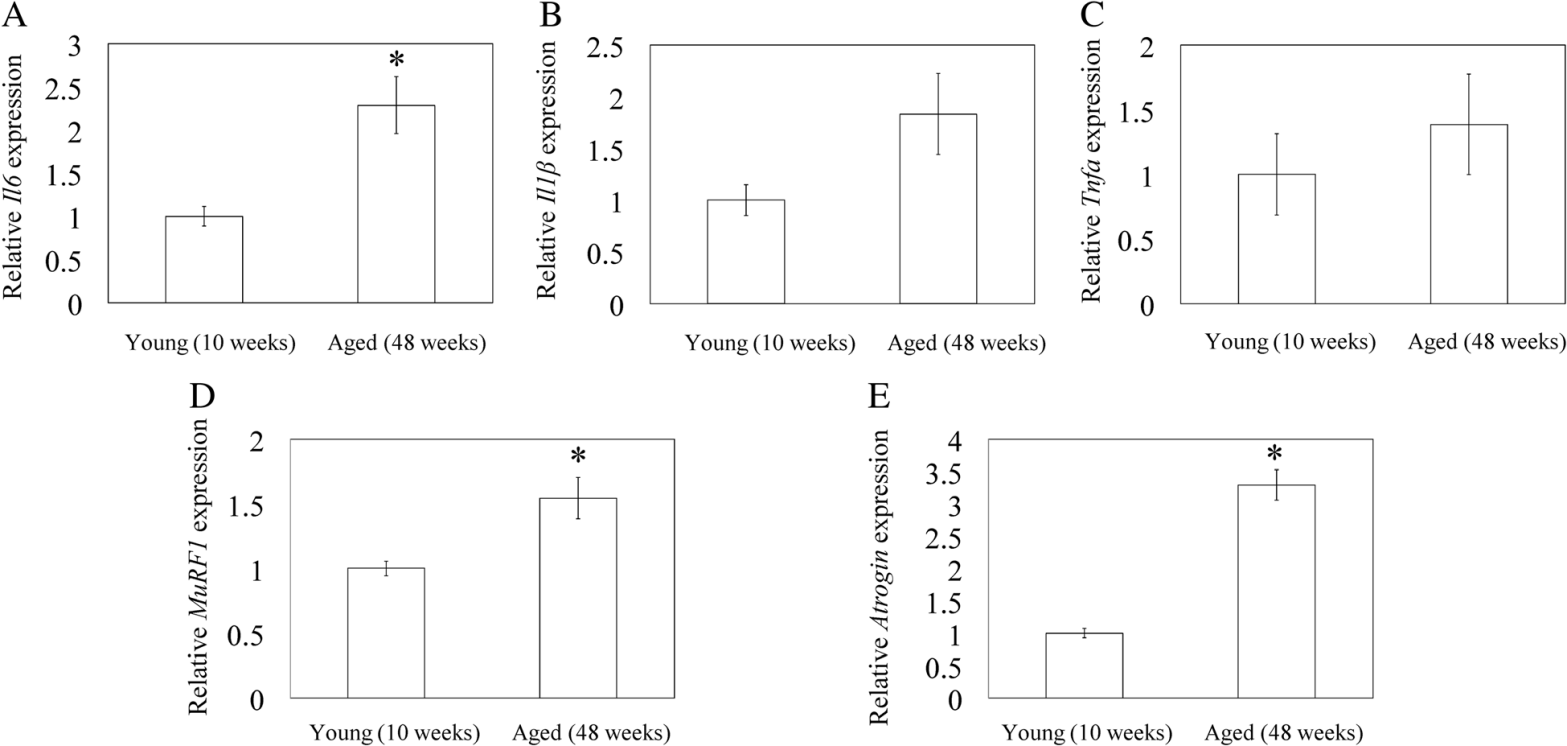
Quantitative reverse-transcription polymerase chain reaction analysis of inflammatory cytokines and muscle breakdown-related gene expression in quadriceps muscles. Relative gene expression of inflammatory cytokines, *Il6* (**a**), *Il1b* (**b**), *Tnfa* (**c**), and muscle breakdown-related gene *Atrogin1* (**d**) and *MuRF1* (**e**) in quadriceps muscle extracted from young (10 weeks) and aged (48 weeks) rats. Data represent mean ± SE (*n* = 10). * *p* < 0.05, between young and aged groups

To evaluate ectopic inflammation and muscle breakdown in skeletal muscle, we performed Q-RT-PCR to examine changes in the gene expression of inflammatory cytokines, *Il1b, Il6 and Tnfa*, in quadricep muscle. The aged group had significantly higher *Il6* mRNA expression than the young group (*p* = 0.001; Fig. 1a). In contrast, there was no significant difference in *Il1b and Tnfa* mRNA expression (*p* = 0.096 and *p* = 0.327, respectively; Fig. 1b, c). *MuRF1 and Atrogin1* mRNA expression were higher in the quadriceps muscles of aged rats than young rats (*p* = 0.002 and *p* < 0.001, respectively; Fig. 1d, e).

### IMAT

**Fig. 2:**
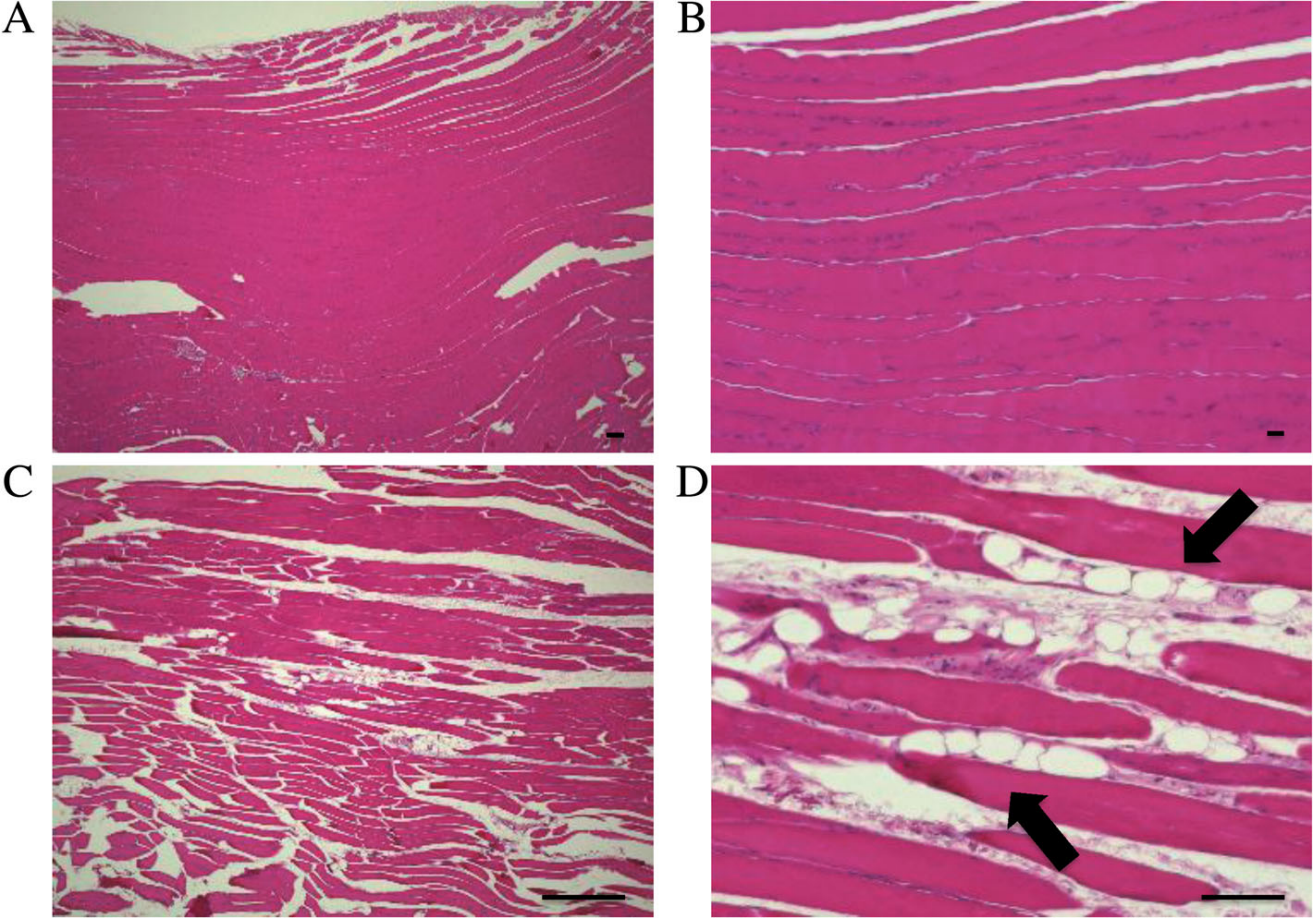
Accumulation of adipose tissue in quadricep muscles. Representative micrographs of muscle sections stained with hematoxylin-eosin from young (10 weeks) and aged (48 weeks) rats. **a** young (× 40), (**b**) young (× 200), (**c**) aged (× 40), (**d**) aged (× 200). Black arrows indicate adipocytes. Scale bar = 100 μm

To investigate changes in the accumulation of adipose tissue due to aging, we examined the extent of IMAT in histologically-stained sections. Quadricep muscles of aged rats (48 weeks) had greater levels of IMAT than that of young rats (10 weeks) (Fig. 2a–d).

### *Leptin* expression and leptin protein concentration in quadricep muscles

**Fig. 3:**
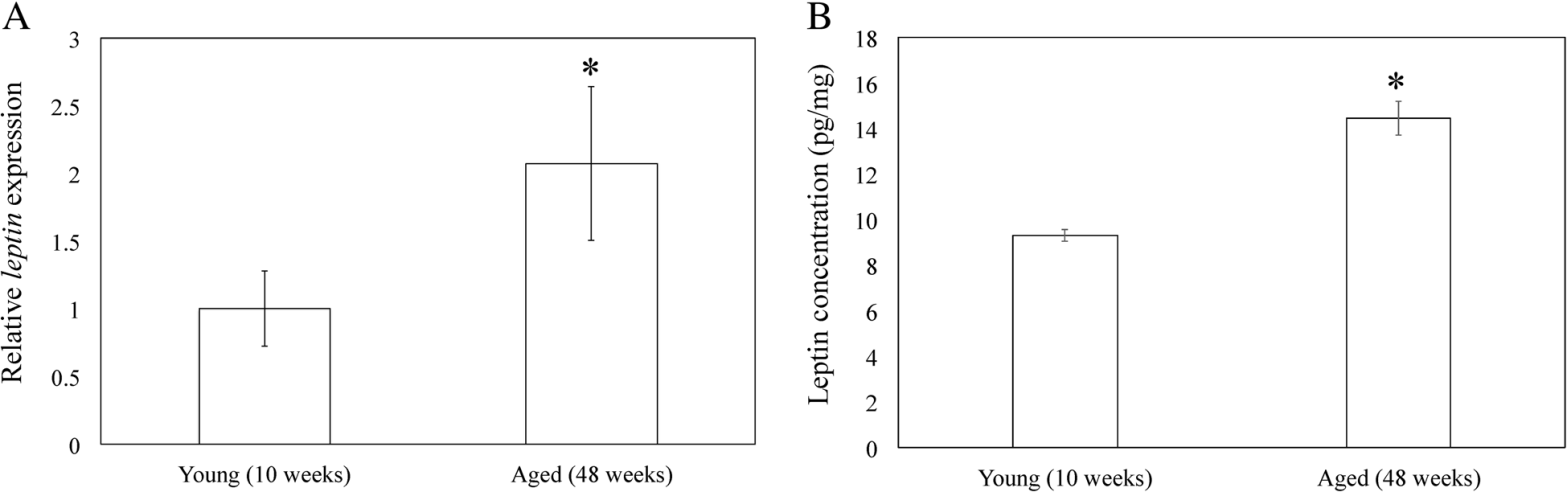
Leptin gene expression by quantitative reverse-transcription polymerase chain reaction analysis and leptin protein concentration by enzyme-linked immunosorbent assay in quadricep muscles. **a** Relative *leptin* gene expression in quadricep muscles extracted from young (10 weeks) and aged (48 weeks) rats. **b** Leptin protein concentration in quadriceps muscles from young (10 weeks) and aged (48 weeks) rats. Data indicate mean ± SE (n = 10). * *p* < 0.05, between young and aged groups

To investigate whether increased IMAT due to aging results in leptin production, we examined *leptin* mRNA expression and leptin protein expression in the quadricep muscles of aged and young rats. *Leptin* mRNA expression and leptin protein concentration were significantly higher in the aged group than in the young group (*p* = 0.049 and *p* < 0.001, respectively; Fig. 3a, b).

### Effect of leptin on *Il1b, Il6* and *Tnfa* expression in quadricep muscle-derived cells

**Fig. 4:**
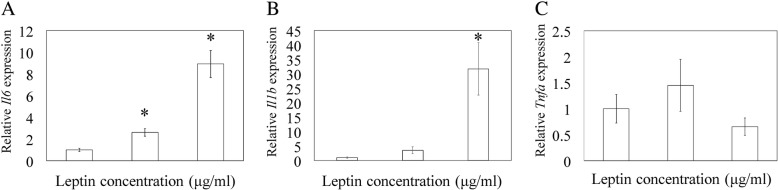
Effect of leptin on *Il1b, Il6,* and *Tnfa* mRNA expression. Effect of leptin on *Il6* (**a**), *Il1b* (**b**), *Tnfa* (**c**) gene expression in cultured quadricep muscle-derived cells. Quadricep muscle-derived cells were stimulated with 0 (control), 1, or 10 μg/mL rat recombinant leptin. Gene expression in the leptin-stimulated groups was compared with that in the control group. Data indicate mean ± SE (n = 10). * *p* < 0.05, compared to control

In vitro experiments were performed to elucidate the relationship between IL-6, IL-1β, TNF-α and leptin. Q-RT-PCR analysis showed that the additional of exogenous leptin at 1 and 10 μg/ml significantly elevated *Il6* mRNA expression compared to control (*p* = 0.001 and *p* < 0.001, respectively; Fig. 4a). *Il1b* mRNA expression was also significantly elevated in the presence 10 μg/ml leptin (*p* = 0.025; Fig. 4b). There was no difference between the leptin-stimulated and control groups in *Tnfa* expression (*p* = 0.279; Fig. 4c).

## Discussion

This study aimed to examine changes to the expression of leptin and inflammatory cytokines due to aging and to determine the relationship between these factors in the rat quadricep muscle. We showed that IMAT, leptin gene and protein expression, *Il6, and MuRF1 and Atrogin1* mRNA expression were higher in the quadricep muscles of aged rats than young rats. In addition, stimulation of muscle-derived cells with exogenous leptin significantly and dose-dependently increased *Il1b* and *Il6* gene expression. To our knowledge, this study is the first to examine leptin and inflammatory cytokine expression due to aging in ectopic muscle.

Previous studies have reported that higher IL-6 concentrations in plasma are associated with lower muscle mass and lower muscle strength in elderly people [6], and that aging is linked to elevated IMAT in the thigh muscle in humans [18, 19]. Additionally, IMAT within the fascia is correlated with *Il6* expression in subcutaneous adipose tissue in elderly men [20]. However, these studies analyzed subcutaneous or serum levels of inflammatory cytokines. Here, we showed that IMAT, *Il6,* and muscle breakdown-related gene (*MuRF1*, *Atrogin1*) expression were increased in the quadricep muscles of aged rats compared to young rats. An experimental study showed that IL-6 administration caused muscles to break down in rats [21]. IL-6 stimulate *Atrogin1* mRNA and Atrogin1 protein expression in mice gastrocnemius muscle [22]. Inhibition of Il-6 suppresses *MuRF1* expression and ameliorates tail suspension-induced skeletal muscle atrophy [23]. Taken together, these findings suggest that changes in *Il6* expression with age are associated with the formation of micro inflammation environments in ectopic muscle and a reduction in muscle mass.

Serum leptin is positively associated with IMAT in humans [20]. In our study, an increase in IMAT corresponded with elevated *leptin* expression and leptin protein concentrations in the quadriceps of aged rats. In addition, leptin stimulated *Il6* and *Il1b* expression in quadricep-derived cells. Plasma leptin levels are increased in individuals with sarcopenia and visceral obesity compared to those with sarcopenia or visceral obesity alone [24]. The development of sarcopenia is correlated with raised serum levels of IL-6, an inflammatory factor [7]. Further, IL-1β impaired myoblast differentiation in the murine myoblast cell line C2C12 [25]. Taken together, our findings and those of previous reports suggest that IMAT-derived leptin induces ectopic inflammation through IL-6 and IL-1β and may contribute sarcopenic pathology.

Several studies showed that TNF-α level increases with aging [26, 27]. In our study, there was no difference between 10- and 48-week-old rats in *Tnfa* expression level. We excluded 96-week-old rats from evaluation to eliminate the possible effect of tumor. However, our preliminary experiment showed that *Tnfa* expression in 96-week-old rats was 2.0-hold higher than that in 10-week-old rats. Further investigation using older rats may reveal whether the elevation of *Tnfa* in skeletal muscle contributes to sarcopenic pathology.

Two limitations of this study warrant mention. First, we performed in vitro experiments using cells derived from young rats in standard culture conditions. Leptin resistance was introduced by negative regulators of leptin signaling such as inflammatory signals, including IKKβ/NFκB and ER stress [28, 29]. However, to mimic leptin resistance in vivo, a specific condition was needed in vitro [30]. Further investigation under specific conditions using aged rat-derived cells is needed to reveal leptin resistance in aged rats. Second, we investigated only two time points. A better understanding of the development of sarcopenia requires analysis of multiple time points.

## Conclusions

In conclusion, IMAT and *leptin* and *Il6* expression increase with age in rat quadriceps. Our results suggest that IMAT-derived leptin regulates *Il6* expression and creates a micro inflammatory environment in ectopic muscle due to aging.

## Additional file

Additional file 1: **Figure S1**. Age-related changes in *Leptin* mRNA expression. We investigated age-related changes in *Leptin* mRNA expression in quadriceps muscle of rats aged 10, 24, 48, and 96 weeks using quantitative reverse-transcription polymerase chain reaction (Q-RT-PCR) analysis (each *n* = 5). Q-RT-PCR analysis indicated that leptin mRNA expression in 48- and 96-week-old rats was higher than that in 10-week-old rats. (TIFF 242 kb)

## Abbreviations

ELISA: Enzyme-linked Immunosorbent assay
HE: Hematoxylin-eosin
IL-1β: Interleukin-1β
IL-6: Interleukin-6
IMAT: Intermuscular adipose tissue
Q-RT-PCR: Quantitative reverse-transcription polymerase chain reaction
SD: Sprague dawley
SE: Standard error
