# Supplementary figures and images for: Elevated leptin levels induce inflammation through IL-6 in skeletal muscle of aged female rats
Source: BMC Musculoskelet Disord. 2019 May 10;20:199. doi: 10.1186/s12891-019-2581-5 (PMC6511122; doi:10.1186/s12891-019-2581-5)

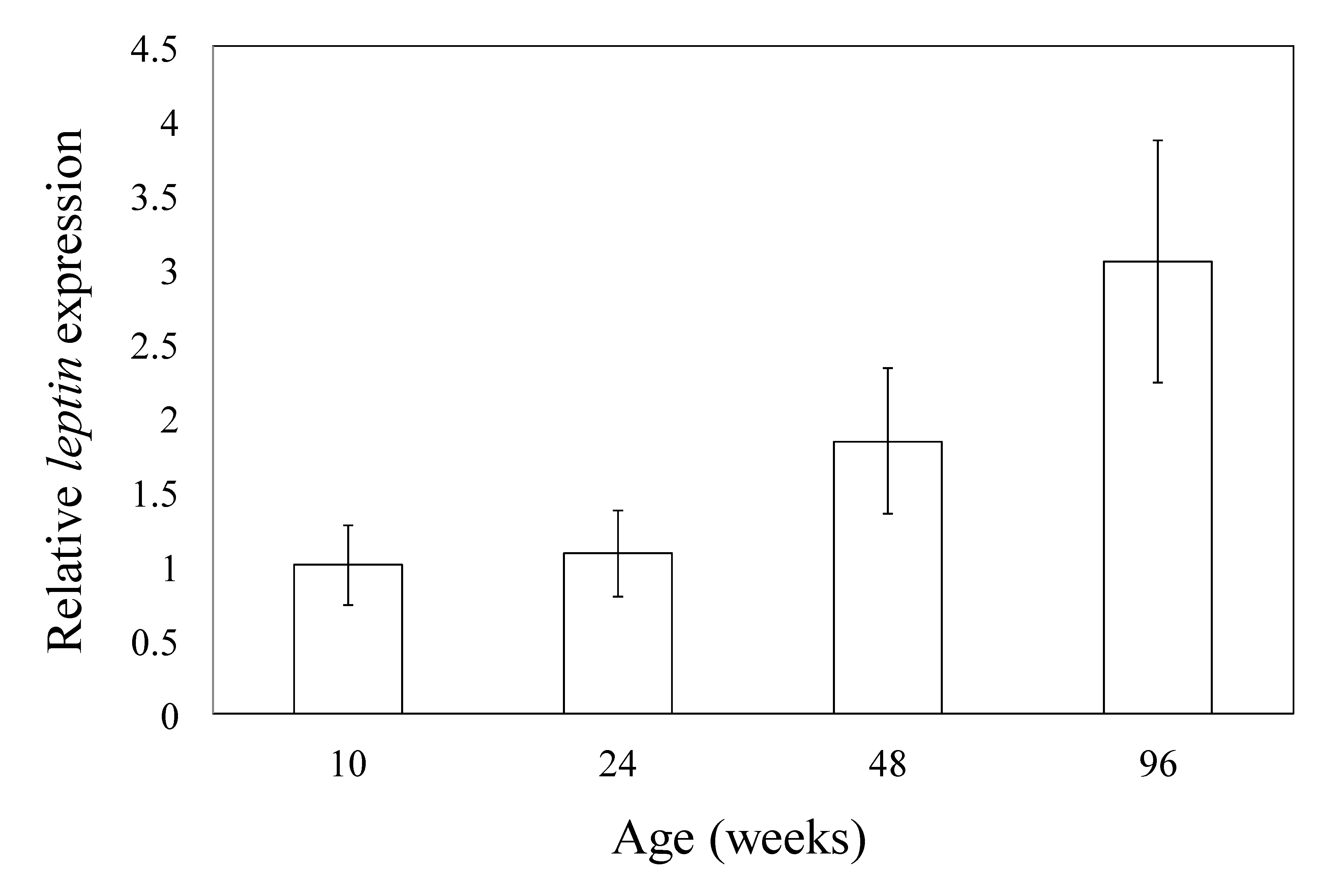

Supplement: Supplementary file 1 — Figure S1. Age-related changes in Leptin mRNA expression. We investigated age-related changes in Leptin mRNA expression in quadriceps muscle of rats aged 10, 24, 48, and 96 weeks using quantitative reverse-transcription polymerase chain reaction (Q-RT-PCR) analysis (each n = 5). Q-RT-PCR analysis indicated that leptin mRNA expression in 48- and 96-week-old rats was higher than that in 10-week-old rats. (TIFF 242 kb) [file 12891_2019_2581_MOESM1_ESM.tiff]
